# Myocarditis as the first sign of SLE: a case report and review of the literature

**DOI:** 10.1007/s00296-026-06238-6

**Published:** 2026-07-04

**Authors:** Chrysanthi Staveri, Sophie I. Mavrogeni, Alexandros Alexopoulos, Panagiotis Chronopoulos, Stamatis-Nick C. Liossis

**Affiliations:** 1 Division of Rheumatology, Department of Internal Medicine, Patras University Hospital, 26504 Patras, Greece; 2 Aghia Sophia Children’s Hospital, University Research Institute of Maternal and Child Health, National and Kapodistrian University of Athens School of Medicine, 11527 Athens, Greece; 3 Patras General Clinic ’‘OLYMPION S.A’’, 26443 Patras, Greece; 4 Emergency Department, Patras University Hospital, 26504 Patras, Greece; 5 Division of Rheumatology, Department of Internal Medicine, Patras University Hospital, 26504 Patras, Greece; 6 Division of Rheumatology, University of Patras School of Medicine, 26500 Patras, Greece

**Keywords:** Myocarditis, Lupus erythematosus, Systemic, Magnetic resonance imaging, Heart failure

## Abstract

Lupus myocarditis (LM) is a rare but potentially life threatening manifestation of systemic lupus erythematous (SLE). LM may be rarely the initial presentation of SLE or ma y be presented during the course of the disease. Herein, we report a case of an adult female patient who presented with a 3-day history of abdominal pain, fever and recent onset dyspnea on exertion. Twenty-four hours later the patient developed acute heart failure went into cardiac arrest during her transfer from the internal medicine department to the CT scanner. She was intubated, an intra-aortic balloon pump was inserted, and was transferred to the intensive care unit (ICU). Based on clinical signs, laboratory parameters and cardiac imaging and after the exclusion of other causes of acute heart failure LM was diagnosed. The patient received high doses of corticosteroids and cyclophosphamide and she was improved. The vasopressors were tapered off and the intra-aortic balloon pump was removed. The patient improved and she shifted out of the ICU. The initially increased *t*roponin levels were decreased, while the ejection fraction increased to 60% five days later. The patient was followed as an outpatient after hospital discharge. At 6 months of follow-up the she was on treatment with low doses of oral steroids, hydroxychloroquine and azathioprine and her ejection fraction is 73% without signs of active disease. No recurrences of her LM have been observed during her 32 month follow-up. Prompt recognition and aggressive immunosuppressive treatment may lead to favourable prognosis.

**Supplementary Information:**

The online version contains supplementary material available at 10.1007/s00296-026-06238-6.

## Introduction

Systemic lupus erythematous (SLE) is an autoimmune disease with clinical phenotypes ranging from mild mucocutananeous manifestations to life**-**threatening (multi)organ involvement. The heart is reportedly affected in more than 50% of the patients with SLE [1]. Any structure of the heart can be involved including the pericardium, myocardium, endocardium, coronary arteries, cardiac valves as well as the conduction system [2]. Lupus myocarditis (LM) is a rare but potentially life-threatening manifestation of SLE occurring in 5–10% of patients [3]. Autopsy studies have demonstrated evidence of subclinical myocarditis in up to 30–60% of the patients with lupus [4]. Myocarditis as an initial presentation of SLE has been reported in children but it is an extremely rare initial presentation in adults [5]. A two-stage pathogenesis model for the development of LM has been recently proposed [6]. In the first stage, heart damage may expose nuclear antigens activating autoantibodies and autoreactive T cells. In the second stage, tissue-resident memory T cells of the heart are activated during disease flares leading to the development of LM.

### Case presentation

A 34-year-old woman with no significant past medical history was admitted with a 3-day history of abdominal pain, fever and recent onset dyspnea on exertion. Physical examination was remarkable only for abdominal right upper quadrant tenderness. Abnormal laboratory parameters at patient presentation included: WBC: 14,770/mm^3^, Hb: 10.4 g/dL, elevated liver enzymes (SGOT: 922 U/l, SGPT: 757 U/l), CRP:17.3 mg/dL (< 0,8). Coagulation tests were normal (aPTT: 24.6 s). An electrocardiographic tracing illustrated the negative T-wave in the V2-V6 precordial leads. Gallbladder wall and periportal oedema as well as pan-serositis were seen in the Computed Tomography (CT) scans of the abdomen and empirical treatment with antibiotics (ceftriaxone and metronidazole) was initiated. Twenty-four hours later she developed acute heart failure. The high-sensitive troponin levels were 102.3 pg/ml (0–15.6). Trans-thoracic echocardiogram demonstrated generalized wall hypokinesia with an ejection fraction (EF) of 35%, Global Longitudinal Strain (GLS): −6.3% and moderate right ventricular dilatation (Supplementary Videos A: Transthoracic Echocardiogram of the patient before treatment demonstrating global hypokinesia, reduced LVEF (35%), GLS − 6.3% and moderate right ventricular dilatation (Parasternal long axis, Parasternal short axis, Apical 4 chamber and Subcostal views). The patient went into cardiac arrest during her transfer from the internal medicine department to the CT scanner. She was intubated, an intra-aortic balloon pump was inserted, and was transferred to the intensive care unit (ICU). Left heart catheterization did not illustrate coronary atherosclerotic disease or cardiac tamponade, while CT Pulmonary Angiogram ruled out a pulmonary embolism. Upon exposure to sunlight through the window in the ICU a butterfly rash and an inverse Gottron’s sign appeared. Further work-up revealed that the patient tested positive for ANA (1: 640, speckled pattern) and also positive for anti-RNP autoantibodies with low complement C3 and C4 levels (60 mg% and 7 mg%, respectively). The patient met the 2019 European League Against Rheumatism and American College of Rheumatology classification criteria for SLE (Total score: 17; fever:2, acute cutaneous lupus:6, pleural and pericardial effusion: 5, low C3 and low C4:4) [11]. Despite the fact that our patient tested positive for anti-RNP there was no evidence, such as Raynaud phenomenon, puffy hands, synovitis or interstitial lung disease suggesting mixed connective tissue disease or an overlap syndrome.

**Fig. 1:**
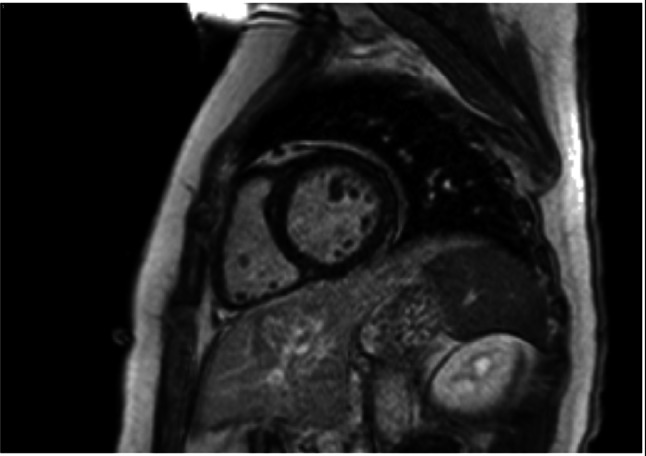
Cardiac MRI short axis view demonstrating epicardial late gadolinium enhancement located mainly in the lateral wall, consistent with non-ischemic injury, most likely myocarditis. There is also associated small pericardial effusion

**Fig. 2:**
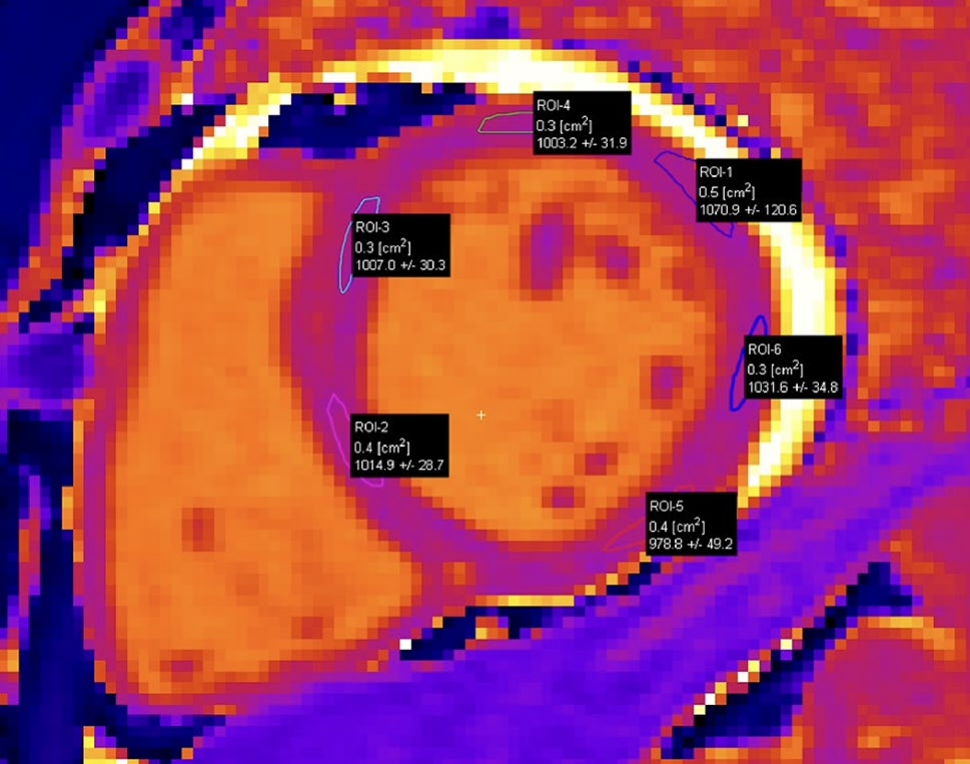
TT1 mapping of the short axis slice shows increased native T1 relaxation times in the lateral wall. This quantitative mapping supports the presence of myocardial injury complementary to the late gadolinium enhancement findings

**Fig. 3:**
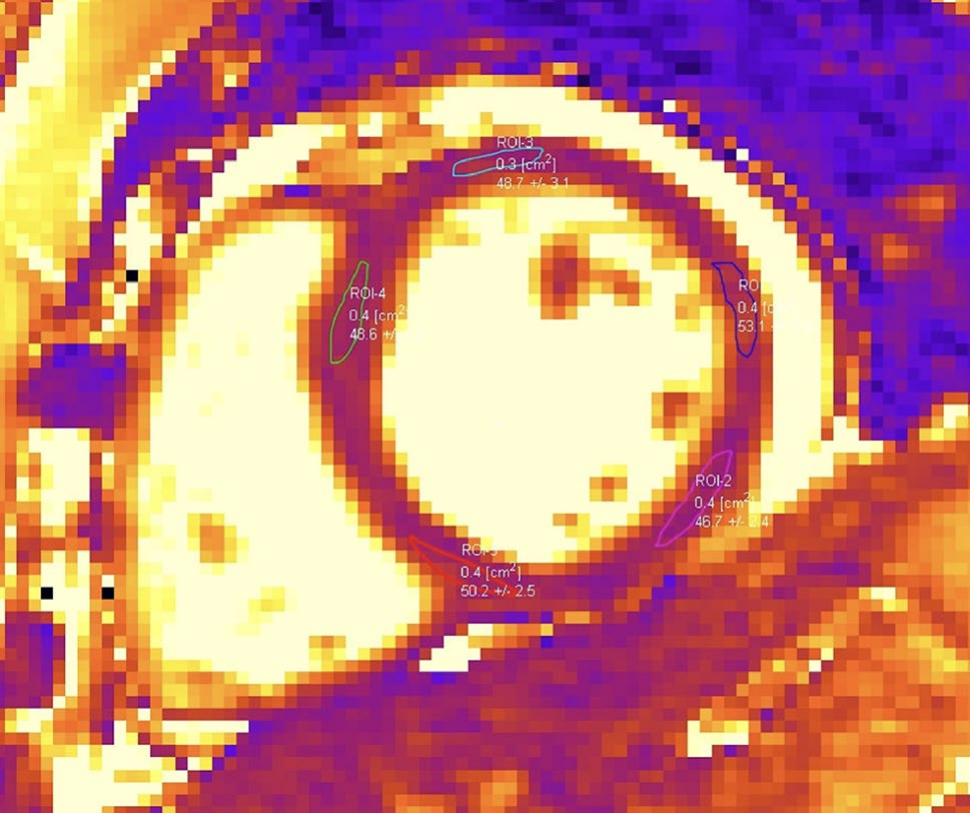
T2 mapping of the short axis slice revealing slightly elevated T2 values in focal segment of lateral wall, indicative of limited residual myocardial edema / inflammation during the recovery phase

A panel for viruses was negative (HIV, CMV IgM, Rubella IgM, Toxoplasma, EBV, CMV, Leishmania, Leptospira, Mycoplasma Pneumoniae, Borrelia). The quantitative immunoglobulin assay revealed IgG: 2390 mg/dl, IgA: 364 mg/dl, IgM: 384 mg/dl. Anticardiolipin IgG and anti-β2 glycoprotein 1 IgG and IgM antibodies were negative. A cardiac magnetic resonance imaging (CMR) showed epicardial and enhancement in the lateral and lower walls and of mild intensity in the inter ventricular septum mainly in the posterior with signs of inflammation (swelling) in the above areas (Figs. 1, 2 and 3).

SLE was diagnosed and IV pulsed 1 g of methylprednisolone and 1 g of cyclophosphamide (CYC), followed by 1 mg/kg/d oral prednisone and hydroxychloroquine were administered. The vasopressors were tapered off and the intra-aortic balloon pump was removed. Other medications included fondaparinux 2.5 mg daily and bisoprolol 2.5 mg daily. The patient improved and she shifted out of the ICU. The initially increased *t*roponin levels were decreased to 30.7 pg/ml and 17 pg/ml at 5 and 10 days after treatment. Her EF increased.

to 60% five days later, GLS:−19%, right ventricle of normal size (Supplementary Videos B: Transthoracic Echocardiogram of the patient shortly after treatment demonstrating normal LVEF, GLS:−19% and right ventricle of normal size (Parasternal long axis, Parasternal short axis, Apical 4 chamber, Apical 2 chamber, and Apical long axis views) and to 73% on hospital discharge. The patient was followed as an outpatient after hospital discharge. At 6 months of follow-up the she was on treatment with low doses of oral steroids (methylprednisolone 4 mg/d), hydroxychloroquine (HCQ) 400 mg/d and azathioprine 150 mg/daily, her ejection fraction is 73% without signs of active disease. No recurrences of her LM have been observed during her 32 month follow-up.

### Search strategy

A systematic literature search was conducted in Medline/PubMed, Scopus, Web of Science and Directory of Open Access Journals in order to identify English language sources using the following keywords: ‘’lupus myocarditis’’ and ‘’initial manifestation’’. Cases of patients who developed LM during the course of the disease were excluded. This case-based review follows the criteria of the CABARET recommendations of the EQUATOR network [51].

### Literature review

We identified 9 cases of adult patients who developed LM as the initial manifestation of SLE [52−59]. One patient presented with cardiogenic shock [54], while the remaining 7 patients presented with symptoms of dyspnea and/or chest pain. One patient had a coincident large pericardial effusion [59]. Concomitant non-cardiac manifestations of the patients included LN in 2 patients, CNS involvement in one patient and acute liver injury in another one patient [52, 57]. CMR was performed in 3 patients [53, 55, 59] and an endomyocardial biopsy (EMB) in 2 patients [53, 55]. All patients were improved after the introduction of immunosuppressive treatment. One patient developed atrial fibrillation during his follow-up [56].

## Discussion

LM is most commonly diagnosed in young patients with lupus with a median age of 33 years and early in the disease course (median disease duration 2.7 years) [7]. LM as the initial presentation of SLE has been observed in 0.37% in a lupus cohort [7], whereas as a late manifestation is extremely rare. Before the introduction of corticosteroids in the treatment of SLE, LM was observed in 80% of the patients with SLE on autopsy studies [9]. However, since the management of SLE was improved, the prevalence of LM was reduced to 6% [10]. The mortality rates range between 10.3% and 23.3% [7, 8].

The diagnosis of LM is based on a constellation of the clinical features of myocardial dysfunction supported by imaging findings that involve myocardium in a non-coronary artery distribution after exclusion of other causes of myocardial involvement. The differential diagnosis of myocardial involvement in patients with SLE includes ischemia due to accelerated atherosclerosis, coronary artery thrombosis or coronary angiitis, LM, viral myocarditis and HCQ cardiotoxicity. In cases of HCQ-induced cardiotoxicity symptom inception may occur at a median time of 10 years after treatment initiation [12].

LM exhibits a broad clinical spectrum ranging from resting tachycardia to overt congestive heart failure and cardiogenic shock. Other symptoms include dyspnea on exertion and palpitations. Pleuritic chest pain is a complain when concomitant pericarditis is present. Ischemic-type chest pain is a common feature of a viral myocarditis and not of LM.

There are distinctive features that distinguish patients with LM from patients with myocarditis or SLE alone [13]. Presence of anti-β2-glycoprotein I antibodies, antiphospholipid syndrome and involvement of a higher number of British Isles Lupus Assessment Group (BILAG) domains in patient history were significantly associated with LM. Additionally, some patients with LM may have concomitant lupus nephritis (LN) [49, 50]. CMR findings suggesting subclinical myocardial involvement are more pronounced in patients with lupus compared to SLE patients without LN [14]. On the other hand, central nervous system involvement and treatment with antimalarials were negatively associated with the risk of primary cardiac disease including myocarditis in patients with SLE [15].

Among serological markers autoantibodies that have been more strongly associated with LM include anti-Ro (SS-A), anti-RNP and anti-dsDNA [16, 18, 39]. Biomarkers of cardiac injury such as troponin and creatine kinase may be increased during LM, although they lack specificity. Of note, highly-sensitive troponin is associated with CMR evidenced subclinical myocarditis in patients with SLE [45].

EMB is considered to be the gold standard for the diagnosis of LM, but with limited clinical application. A case series study of EMBs in patients with SLE and suspected myocardial involvement illustrated that nonspecific interstitial fibrosis and myocyte hypertrophy were the most common findings and only 3 out of 11 patients showed evidence of a mild myocardial inflammatory infiltration [19]. In addition, Mavrogeni, et al. reported that the sensitivity of EMB to detect LM was < 50% (3/7) in SLE patients with clinical and CMR findings consistent with LM [20]. However, EMB may be useful for the exclusion of other causes of myocardial involvement.

Echocardiography is a classical method used to support the diagnosis of LM. The most common echocardiographic findings include pericardial effusion, reduced LVEF, global hypokinesis and regional wall motion abnormalities in a non-arterial distribution. Strain analysis may detect early LV dysfunction in patients presented with a preserved LVEF (> 50%) [4].

CMR is considered as the non-invasive method of choice for the diagnosis of LM [13, 22–28, 42]. The hyper enhancement areas (LGE) in LM are characterized as non-ischemic, they have a subepicardial or intramyocardial distribution and they do not follow the distribution of coronary arteries. The same type of lesions may also be observed in patients with antimalarial-induced cardiomyopathy [21]. In addition, some CMR imaging findings may overlap with viral or other inflammatory cardiomyopathies; evaluation from an expert cardiologist would be of a great value. CMR can also exclude the presence of microvascular coronary artery disease, which may be presented with a similar clinical scenario. In this case the invasive coronary angiography is normal as in myocarditis. However, the CMR shows areas of subendocardial LGE either localised or diffuse indicative of coronary microvascular disease.

Positron emission tomography/(PET)/CT is another non-invasive method in the investigation of patients with LM and especially in those with diminished renal function or metal implants [29].

The treatment of LM typically involves a combination of glucocorticoids and immunosuppressants along with heart failure management [41], since no randomised controlled trials have been conducted. Treatment includes prednisone (0.5–1 mg/kg) IV for three days followed by a daily oral use with tapering and CYC or mycophenolate mofetil [30, 31]. Of note, it has been proposed that treatment with CYC should be reserved for patients presented with severe disease corresponding to a low LVEF [8]. In resistant cases of LM rituximab and intravenous immunoglobulin (IVIg) may be considered [32–34]. Patients with severe and life-threatening LM may also benefit from plasmapheresis or immunoadsorption [35–37]. A study established the crucial role of the aggressive immunosuppressive treatment of patients with SLE in reducing the odds of LM [17].

Data regarding the long-term outcome of patients with LM are based on case series and generally relapses are rare [38]. Residual myocardial dysfunction corresponding to an LVEF ≤ 50% has been recorded in almost 21% of the patients [7]. Moreover, it has been demonstrated that LM did not significantly affect disease activity over time, however did affect at least partially damage accrual and also survival of patients with SLE, particularly those with ≥ 5 years disease duration [3].

## Conclusion

Initial manifestations of SLE are highly variable and may mimic other entities, making the diagnosis challenging [48]. We describe a case of a young female patient who presented with life threatening LM. The acute and rapidly progressive presentation of heart failure in a young patient without any of the traditional risk factors for the development of CVD represents a medical emergency. After the exclusion of myocardial infarction and pulmonary embolism, other causes should be considered. The diagnosis was based on a constellation of clinical findings, imaging modalities especially CMR and laboratory parameters and after the exclusion of other potential causes. A potential limitation of our case report is the fact that EMB was not performed. Furthermore, our patient improved after the administration of pulsed methylprednisolone and CYC.

Unusual manifestations of newly diagnosed SLE reflect the high clinical heterogeneity of the disease. A case of a 23-year old male patient who presented with acute pancreatitis, enteritis and acute liver failure has been reported. The diagnosis was made after the exclusion of other causes, detection of positive ANA, anti-dsDNA and hypocomplementemia, an histologically confirmed LN and response to glucocorticoids and immunosuppressive treatment [46]. Treatment in our patient was successful but resistant cases have been reported. More specifically, an 18-year-old male patient was diagnosed with SLE, when he had presented with right femoropopliteal deep vein thrombosis, autoimmune haemolytic anemia and thrombocytopenia, while he tested positive for anti-dsDNA autoantibodies and anti-cardiolipin antibodies. At 6 years after the diagnosis he developed LM. The patient did not respond to treatment with glucocorticoids and cyclophosphamide, but he was improved after the introduction of IVIg [47].

Early recognition and appropriate treatment of SLE patients at increased CVD risk might reduce morbidity and mortality [40, 44]. To this point, CMR has become a valuable tool in the early identification of structural or functional disturbances before the onset of clinically overt CVD [43].

## Supplementary Information

Below is the link to the electronic supplementary material.

Supplementary Material 1

Supplementary Material 2

Supplementary Material 3

Supplementary Material 4

Supplementary Material 5

Supplementary Material 6

Supplementary Material 7

Supplementary Material 8

Supplementary Material 9

Supplementary Material 10

Supplementary Material 11

Supplementary Material 12

Supplementary Material 13

Supplementary Material 14

## Data Availability

No new data were created.

## Declarations

### Disclaimer

No part of this case-based review is copied, published elsewhere, or generated by AI.
