# Supplementary figures and images for: Myocarditis as the first sign of SLE: a case report and review of the literature
Source: Rheumatol Int. 2026 Jul 4;46(7):188. doi: 10.1007/s00296-026-06238-6 (PMC13332954; doi:10.1007/s00296-026-06238-6)

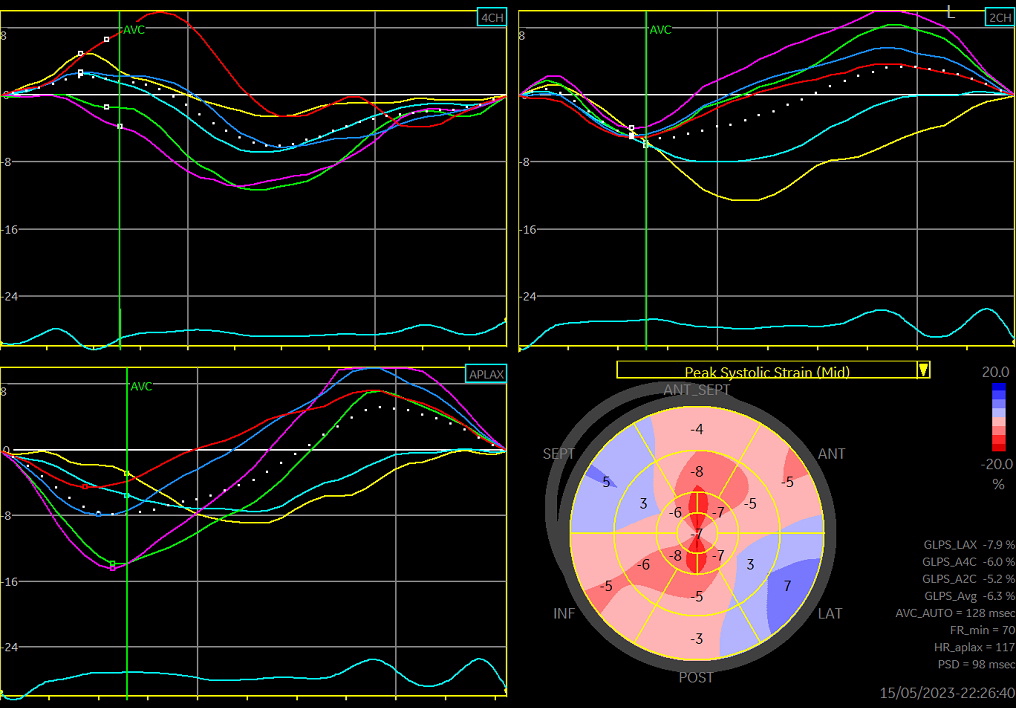

Supplement: Supplementary file 5 — Supplementary Material 5 [file 296_2026_6238_MOESM5_ESM.jpg]

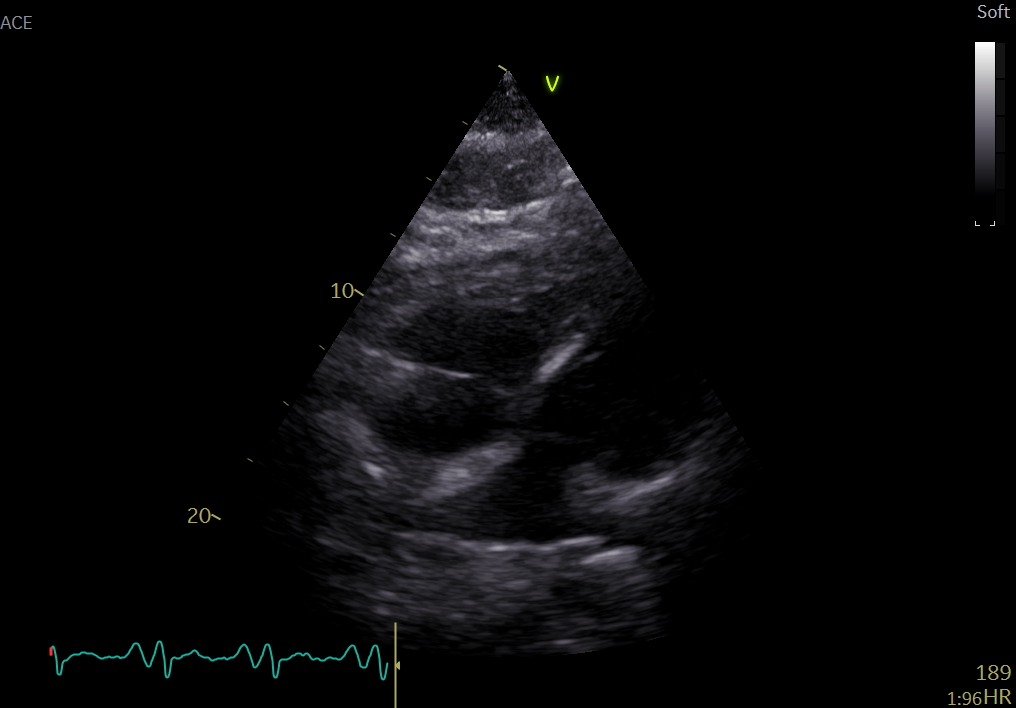

Supplement: Supplementary file 6 — Supplementary Material 6 [file 296_2026_6238_MOESM6_ESM.jpg]

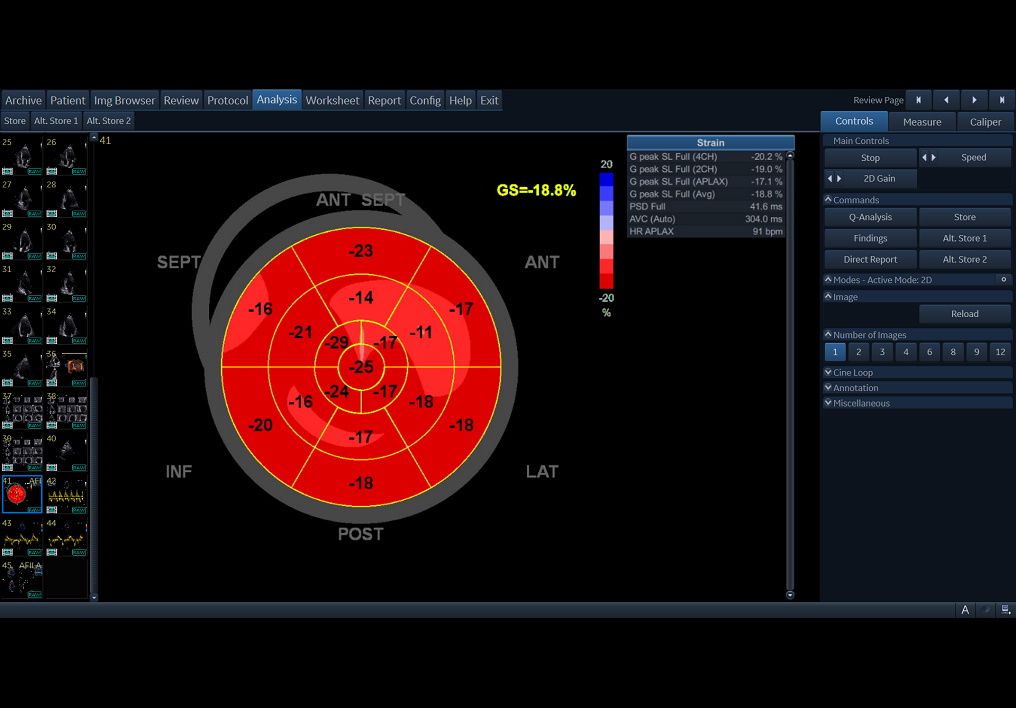

Supplement: Supplementary file 9 — Supplementary Material 9 [file 296_2026_6238_MOESM9_ESM.jpg]
